# Microglial Metabolism After Pediatric Traumatic Brain Injury – Overlooked Bystanders or Active Participants?

**DOI:** 10.3389/fneur.2020.626999

**Published:** 2021-01-25

**Authors:** Aria C. Shi, Ursula Rohlwink, Susanna Scafidi, Sujatha Kannan

**Affiliations:** ^1^Department of Anesthesiology and Critical Care Medicine, Johns Hopkins University School of Medicine, Baltimore, MD, United States; ^2^Neuroscience Institute and Division of Neurosurgery, University of Cape Town, Cape Town, South Africa; ^3^The Francis Crick Institute, London, United Kingdom

**Keywords:** microglia, metabolism, pediatric, brain trauma, energy

## Abstract

Microglia play an integral role in brain development but are also crucial for repair and recovery after traumatic brain injury (TBI). TBI induces an intense innate immune response in the immature, developing brain that is associated with acute and chronic changes in microglial function. These changes contribute to long-lasting consequences on development, neurologic function, and behavior. Although alterations in glucose metabolism are well-described after TBI, the bulk of the data is focused on metabolic alterations in astrocytes and neurons. To date, the interplay between alterations in intracellular metabolic pathways in microglia and the innate immune response in the brain following an injury is not well-studied. In this review, we broadly discuss the microglial responses after TBI. In addition, we highlight reported metabolic alterations in microglia and macrophages, and provide perspective on how changes in glucose, fatty acid, and amino acid metabolism can influence and modulate the microglial phenotype and response to injury.

## Introduction

Traumatic brain injury (TBI) is the leading cause of pediatric trauma death and disability, affecting up to 280 out of 100,000 children worldwide (1). The long-term morbidity of TBI is often difficult to quantify, as TBI can alter many aspects of a young person's development. These long-term morbidities depend heavily on the patient's age at the time of injury, as well as injury severity, and can range from arrested development to deficits in memory and attention that are typically detected in school-age children (2, 3). Sequelae later in life include social and behavioral impairments such as depression, anxiety, and sleep disorders (4, 5).

Despite the devastating consequences of TBI and increasing pre-clinical and clinical research, treatment options are very limited. The vast majority of interventions rely on supportive care for the acute and chronic sequelae of injury. Little has proven to be effective in limiting the tissue and cellular damage that occurs during the primary mechanical injury, nor in diminishing the induced secondary injury that results from ongoing inflammation in the brain. While a degree of initial inflammation in the brain and surrounding tissues is important for immune protection and wound repair (6, 7), failure to revert to baseline or continued dysregulation can perpetuate inflammation that further exacerbates cellular damage (8, 9). This change in the immune response is accompanied by shifts in the metabolic profile that can further perpetuate inflammation in a vicious cycle (10–12). Understanding how these metabolic changes relate to microglial immune dysregulation after pediatric TBI is crucial for identifying cell-specific therapeutic targets to suppress ongoing secondary inflammatory injury. However, research addressing this in TBI and in injuries to the developing brain is limited, and further investigation is warranted. In this article we review what is known about microglial and metabolic alterations after injury to the immature brain, and highlight the need for future research to elucidate the role of metabolic re-programming in modulating microglial immune response in pediatric TBI.

## Microglia in Pediatric TBI

Microglia, the primary immune cells of the CNS, are key instigators of cerebral inflammation and the resultant secondary injury after TBI. Microglia are considered the resident macrophages of the brain: they survey their CNS microenvironment for pathogens, migrate to areas of injury and release inflammatory mediators when injury or infection is detected (8, 13). In the developing brain, microglia play an important role in the brain's normal maturation by performing synaptic pruning, and disruption of microglial activity during development can lead to life-long neurocognitive deficits (7, 14, 15).

Microglial activation after TBI has been observed in both children and adults. Holmin et al. (16) analyzed the inflammatory profiles of human brain tissue biopsies from patients aged 13 to 65 years who underwent surgery for brain contusions after TBI. Biopsies obtained within 24 h after TBI demonstrated an early inflammatory response that was mostly perivascular with margination of neutrophils and expression of pro- and anti-inflammatory cytokines. However, tissue obtained 3–5 days post-injury revealed a more parenchymal distribution, with significant monocyte/macrophage, reactive microglial, neutrophil, and CD4+ and CD8+ T cell infiltration and predominantly pro-inflammatory cytokine expression (16, 17). These observations confirm that although the primary damage after trauma may be mechanical, secondary injury starts a few days later with intense inflammatory activation involving both a local and a peripheral immune response.

Furthermore, clinical evidence shows that secondary injury can cause lifelong changes in microglia. Johnson et al. (18) examined human autopsy brain tissue from patients who had survived a TBI. Those who survived ≥3 months showed extensive, densely packed, reactive microglia in the corpus callosum and adjacent parasagittal cortex, corresponding to areas with ongoing white matter degeneration. Reactive microglia were present in 28% of those who survived more than 1 year and were noted up to 18 years post-TBI. Similarly, Oehmichen et al. (19) examined 305 human brains at the time of autopsy following traumatic closed brain injury (where death occurred between 1 min to 58 years post-injury) from patients aged 1 to 85 years, and demonstrated specific, lifelong changes in microglial (CD68+ stained cells) morphology in these patients.

Although activation and dysregulation of microglia can contribute to secondary injury, microglia are essential for clearing debris and dying neurons during the acute period after injury to permit tissue repair (6). The inflammatory signaling after TBI starts with passive and active drivers of neuroinflammation, provoking microglial activation and response. Microglia express pathogen recognition receptors (PRRs) such as Toll-like receptors (TLRs) and NOD-like receptors (NLRs) that are activated by pathogen-activated molecular patterns (PAMPs) and danger-associated molecular patterns (DAMPs), danger signals secreted by other cells in the CNS (8). Other drivers of neuroinflammation include ATP, glutamate, high-mobility group box 1 (HMGB1), potassium, tumor necrosis factor (TNF), interleukin (IL)-1β, IL-6, monocyte chemoattractant protein 1 (MCP1), and substance P (20). In concert, these molecules signal microglia to change from a quiescent or “normal” morphology to an activated one, which when persistent, contributes to the dysregulated inflammation of secondary injury in TBI. High levels of inflammatory drivers are correlated with worse outcomes after pediatric TBI. In two separate studies that compared children with TBI to age-matched controls (children without TBI who needed lumbar puncture for obstructive hydrocephalus treatment or meningitis rule-out), higher cerebrospinal fluid (CSF) and serum levels of nerve growth factor (NGF), IL-1β, and IL-6 correlated with more severe head injury and worse clinical outcomes (21, 22). Similarly a younger age group is associated with a greater microglial and neuroinflammatory response (23).

The characteristics and functions of microglia activated by an inflammatory milieu have traditionally been described along a spectrum of M1 “classically activated” and M2 “alternatively activated” phenotypes. M1-like microglia are broadly considered pro-inflammatory, perpetuating the inflammatory state; while M2-like microglia are broadly anti-inflammatory, promoting tissue remodeling and matrix deposition (8, 20). However, microglia are not always easily categorized as M1 or M2, as they exhibit transitional fluidity between inflammation, cell proliferation, and remodeling for successful wound repair (24). Nonetheless, the M1/M2 polarization is a good schema for evaluating detrimental microglial activation after TBI, with M1 being a proxy for pro-inflammatory microglia. Both M1 and M2 microglia increase early after TBI, but the M1 phenotype predominates by day 7 post-injury, highlighting dysregulated microglial activation (13, 25). Immune stimuli can lead to specific metabolic programming of microglia and macrophages driving them to an M1 or M2 phenotype. These specific metabolic signatures and alterations and their potential roles in TBI will be discussed in the following sections.

## Metabolic Alterations in Pediatric TBI and Immunometabolism in Microglia

TBI leads to altered metabolism of not only glucose, but also lipids, ketone bodies, and amino acids. These metabolic alterations are particularly relevant in pediatric TBI because the immature brain is especially adept at using alternate substrates to produce energy for brain development (26). Metabolomics studies in rats with TBI have shown changes in several major subgroups of biochemical pathways, most significantly: oxidative phosphorylation, lipid metabolism, the glycolysis, neurotransmitter/neuromodulator metabolism, and amino acid metabolism (10). Comprehensive metabolic profiling of serum samples from 144 hospitalized patients with TBI revealed a handful of metabolite changes that correlated with increasing severity of TBI, including two medium-chain fatty acids (decanoic and octanoic acids) and sugar derivatives including 2,3-bisphosphoglyceric acid (12). Modulating these changes, many of which connect to microglial activation, may help to attenuate secondary inflammatory damage. Here, we review the metabolic perturbations associated with TBI, alongside the known metabolic reprogramming in microglia/macrophages seen in neuroinflammatory disorders, also summarized.

### Glucose Metabolism

The brain has a very high energy demand. The adult brain uses ~25% of all glucose and 20% of all oxygen consumed by the body (26, 27). Glucose is the primary substrate for brain energy and metabolism, but under certain conditions the brain can utilize alternative substrates (e.g., ketones) for energy and metabolism (28). Adult pre-clinical and clinical studies demonstrated that TBI results in up-regulation of glycolysis and pentose phosphate pathway (29–31). However, to date, PPP activity has not been assessed in the developing brain after TBI. Additionally, glycogen storage in the brain may be protective after TBI because astrocyte-mediated glycolysis breaks glycogen into glucose to transiently maintain local glucose levels, but these stores are limited and short-lived (32). Lactate is a product of glycolysis and another potential post-TBI fuel source (31, 33). The healthy neonatal brain uses lactate as an oxidative substrate to supplement energy production as the brain matures; however, controversy remains over whether administering lactate after TBI is beneficial for maintaining glucose metabolism (26, 34).

**Figure 1:**
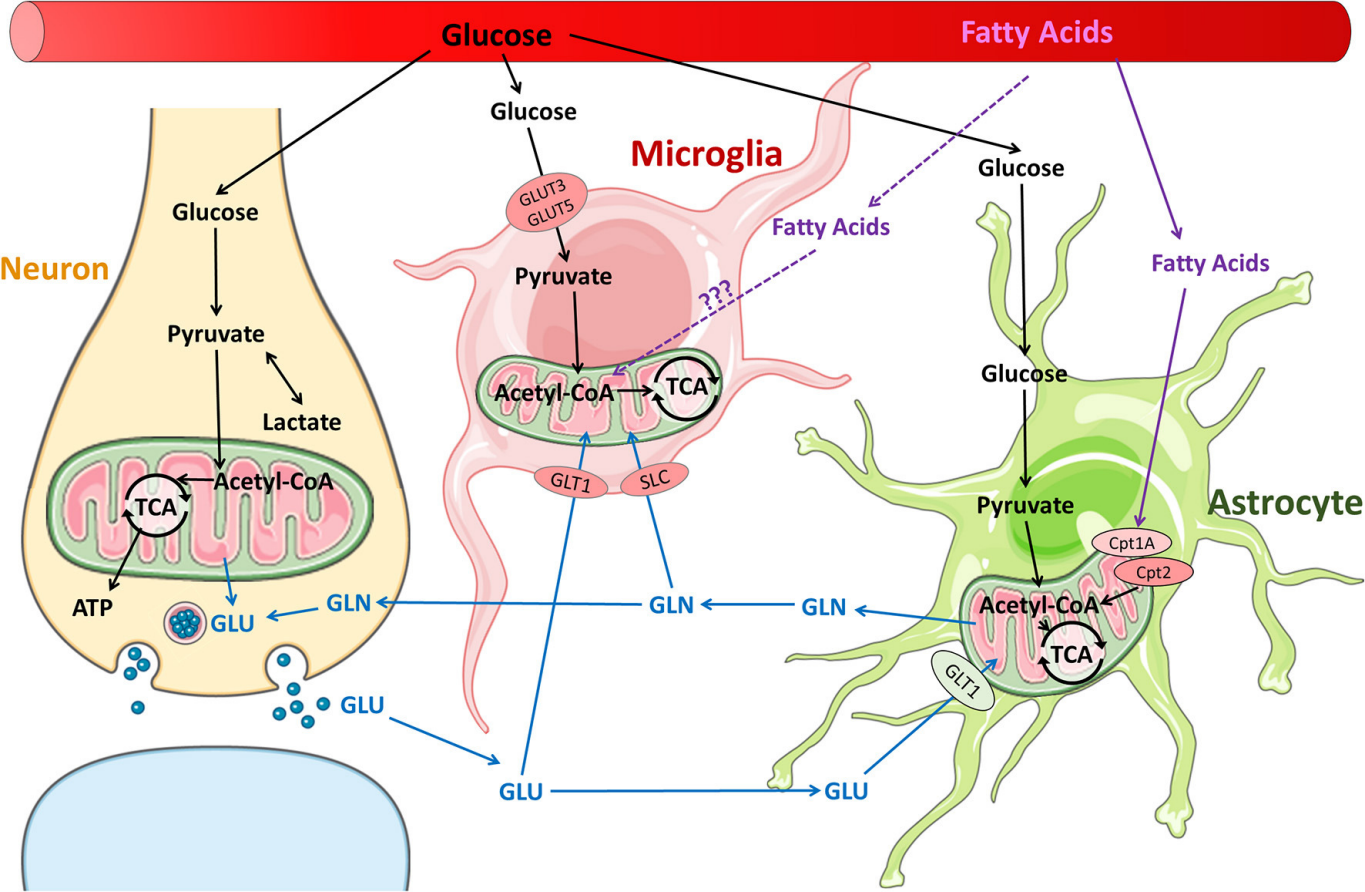
Metabolic pathways in neurons and glia in the normal brain. Glucose is the primary substrate for brain energy and metabolism, and under normal conditions, glucose is utilized by neurons and glia for energy and neurotransmitter synthesis. It is well-established that fatty acids are readily metabolized by astrocytes. It remains unclear whether fatty acids are metabolized by microglia, although fatty acid metabolism has been reported in macrophages.

Microglial surveillance and activity are thought to contribute significantly to the energy demand of the brain (35). Increased microglial activity and proliferation has been extensively studied in both adult and immature brains after TBI; however, what fuels these processes remains unknown. Macrophage and microglial response to neuroinflammation and other neuroinflammatory diseases may offer some insights (36). It is well-established that pro-inflammatory macrophages shift glucose metabolism toward glycolysis and away from oxidative phosphorylation to generate ATP, similar to the “Warburg effect” in the tumor environment. New data support the idea that pro-inflammatory microglia, which express glucose transporters GLUT3 and GLUT5, undergo a similar shift (36, 37) (Figure 1). *In vitro* studies show that pro-inflammatory microglia alter their mitochondrial metabolism in a nitric oxide-dependent manner (37), increasing lactate production, reducing mitochondrial O_2_ consumption, reducing ATP production, and increasing PPP induction (38). Blocking the glycolytic pathway of primary microglia inhibits NF-κB, reduces TNF-α and IL-6 production, and leads to cell death (37). Conversely, culturing primary microglia in increased glucose concentrations increases TNF-α secretion (37). Furthermore, anti-inflammatory stimuli such as IL-4 decrease glucose consumption and lactate production, but increase O_2_ consumption rate, basal respiration, and ATP production. Pro-inflammatory M1 microglia likely upregulate glycolysis and downregulate oxidative phosphorylation, similar to their macrophage counterparts. Although glycolysis is inherently less efficient in ATP generation than oxidative phosphorylation, it is very rapidly activated. Immune cells such as macrophages and microglia that undergo rapid activation by stimulation of PRRs or TLR4s undergo increased glycolysis in order to carry out their effector functions of phagocytosis and inflammatory cytokine production. In macrophages, one of the glycolytic enzymes, hexoskinase1, is known to activate NLRP3 inflammasome which is reportedly increased following TBI in children and infants (39). Increased glycolysis leads to increased production of the intermediate glucose-6-phosphate that is redirected to the oxidative part of the pentose phosphate pathway (PPP) in M1-like microglia/macrophages, leading to increased ROS formation through production of NADPH and NADPH oxidases (NOX) (40). After TBI, NOX2 is upregulated in microglia and macrophages (41, 42). An increase in PPP flux is seen early in adult brains after TBI and at baseline in the healthy neonatal brain. It is not clear whether the PPP is also upregulated in microglia after TBI or whether these changes (increased glycolysis, PPP) are maladaptive or protective in the presence of neuronal and astrocytic dysfunction and cell death.

Microglial glucose metabolism has been better studied in Alzheimer's disease (AD) models than in TBI. In a mouse model of AD, neuroinflammation caused by Aβ amyloid accumulation directly triggered microglia to decrease oxidative phosphorylation and increase glycolysis through the mTOR-HIF-1α pathway (37). Glycolysis can be up-regulated by IFN-γ, a regulator of the mTOR pathway (37). Similar to TBI, AD microglia undergo a shift in morphology from ramified (resting) to amoeboid (active) as the disease progresses (37). Neuroinflammation during AD may lend insight into glucose metabolism of inflammatory microglia among patients with TBI.

M1 and M2 macrophages demonstrate differences in the tricarboxylic acid (TCA) cycle. M2 macrophages and microglia have an intact TCA cycle that is coupled to oxidative phosphorylation (43, 44). However, in M1 microglia/macrophages, the TCA cycle is perturbed at the level of citrate and succinate. Increased citrate generated by the M1 microglia/macrophages is transported out and leads to the formation of fatty acids (44). Excess citrate also promotes the formation of nitric oxide and prostaglandins by activated microglia/macrophages (44–46). Accumulated succinate can lead to stabilization and activation of HIF1α leading to the sustained production of IL1β thereby perpetuating the inflammatory cascade.

### Lipid and Fatty Acid Metabolism

The brain is a lipid-rich organ and has a high expression of fatty acid transporters and fatty acid synthase, which enables extensive fatty acids uptake from the blood as well as synthesis *de novo* in the brain. Brain fatty acids are necessary for neurogenesis, synaptogenesis, and synaptic activity (34, 47, 48). The products of fatty acid synthase are important branch points for biosynthesis and formation of complex lipids or for energy production. In addition, fatty acid oxidation is carried out by neural progenitor cells and astrocytes (47, 49, 50). The long chain acyl-CoAs are shuttled across the mitochondrial membranes via the carnitine palmitoyl transferases (CPTs), are metabolized into acetyl-CoA by β-oxidation, which subsequently enters the TCA cycle (Figure 1). Both the expression of CPTs and enzymes necessary for mitochondrial fatty acid oxidation appear to be developmentally regulated (47).

Lipids are essential for structural functions such as myelin synthesis, and for signaling functions such as lipid raft formation for neurotransmission. These functions can be disrupted after an injury. TBI alters lipid and fatty acid metabolism (10, 12, 26, 36) and increases demand for synthesis of membrane phospholipids to repair brain structures. Membrane phospholipid degradation after TBI leads to increased levels of free fatty acids (FFAs) in the CSF and is thought to contribute to secondary injury. Indeed, an increase in FFAs after TBI is associated with poor outcomes (36). FFAs can activate TLR4 receptors on microglia and macrophages, promoting and propagating inflammation in the brain. Lysophosphatidic acid (LPA) is another bioactive lipid that is increased in the circulation following TBI (51). Increased expression of the LPA receptor in the immature brain (52) may make it more susceptible to LPA after injury.

Fatty acids and lipids are important drivers of metabolic changes in inflammation. Importantly, fatty acid transport proteins, fatty acid binding proteins, and scavenger receptors necessary for transportation of fatty acids are all expressed in microglia and regulated during both normal development and in pathology. M1 and M2 macrophages have distinct fatty acid and lipid metabolism pathways—whether these are the cause or the effect of polarization remains a point of discussion. Pro-inflammatory M1 macrophages have increased fatty acid synthesis that promotes formation of pro-inflammatory cytokines while alternatively activated M2 macrophages increase fatty acid oxidation and oxidative phosphorylation (36, 53).

Omega-3 fatty acids are widely considered to have antioxidant effects in a variety of metabolic and inflammatory diseases, including cardiovascular, autoimmune, and neurologic diseases. Rats that had undergone TBI and treated with intraperitoneal injections of Omega-3 polyunsaturated fatty acids (ω3-PUFA) exhibited a decrease in activated microglia and reduced expression of inflammatory factors TNF-α, IL-1β, IL-5, and IFN-γ when compared to untreated counterparts (54). Omega3-PUFA exert their anti-inflammatory effects by multiple mechanisms including deacetylation of HMGB1 protein, decreasing NF-kB expression, activating PPAR-γ receptor, inhibiting TLRs, activating G-protein-coupled-receptors (GPCRs), and converting to resolvins and neuroprotectins (54–59). Emerging evidence suggests that G protein-coupled receptors GPR40 and GPR120 are activated by ω3-PUFA, which is upregulated on microglia during cerebral artery occlusion (60). GPR120 or FFA4 (a GPCR for long chain unsaturated fatty acids including ω3-PUFA) has been shown to polarize macrophage phenotype to an M2-like, anti-inflammatory form (61). GPR120 has also shown expression on microglia and is over-expressed in injury and neuroinflammation. Deficiency of ω3-PUFA in the immature brain is associated with impaired microglial function and synaptic pruning (62). Whether ω-3 PUFA exert their neuroprotective effects by activating microglial GRP120 remains to be examined.

Docosahexaenoic acid (DHA) comprises 40% of the ω3-PUFA in the brain. Administration of DHA after TBI can decrease oxidative stress and pro-inflammatory microglia in both pediatric and adult rodent CCI models (63–65). Mice with DHA-sufficient diets had less inflammation and faster motor function recovery than those that were DHA-depleted before CCI, although whether these dietary benefits come directly from changes in microglial activity is controversial (66, 67). Omega3-PUFA supplementation may also enhance microglia phagocytosis of myelin, necessary for removal of myelin debris after TBI.

**Figure 2:**
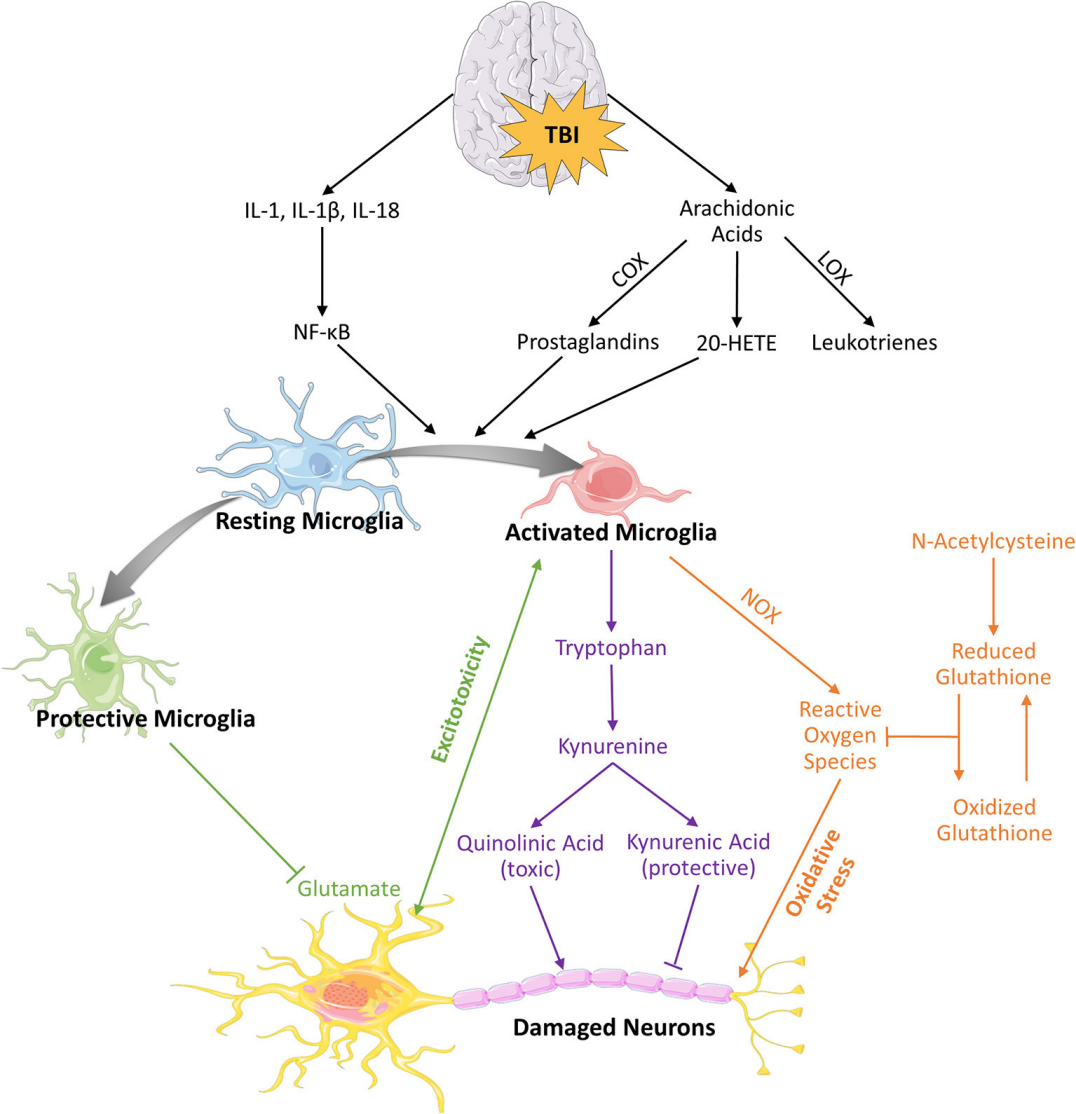
Alterations in amino acids and fatty acids following TBI. Brain injury results in elevated levels of arachidonic acid due to plasma membrane degradation. This results in subsequent cascade of pro-inflammatory metabolites such as leukotrienes, prostaglandins and 20-HETEs, which along with released cytokines lead to activation of microglia and potential recruitment of macrophages. Activated microglia is characterized by upregulation of tryptophan metabolism, increased oxidative stress, and may further propagate neurotoxicity and cell death.

Activated microglia also interact with the arachidonic acid pathway, which makes leukotrienes and prostaglandins—lipids involved in inflammation and tissue repair. TBI induces arachidonic acid release from cell membranes and increases 20-hydroxyeicosatetraenoic acid (20-HETE), a metabolite of the arachidonic acid pathway (Figure 2). Inhibiting 20-HETE synthesis in a rat model of pediatric TBI led to neuroprotection by reducing microglial activation and attenuating proinflammatory cytokines (68). Furthermore, cyclooxygenase (COX) enzymes that catalyze arachidonic acid into prostaglandins are strongly expressed on microglia during neuroinflammation and are upregulated during pathologic conditions such as infection, ischemia, and TBI both in pre-clinical models and in the clinical setting and remain elevated for a prolonged period after the injury (69–71). Additionally, certain polymorphisms of COX-1 and COX-2 have been linked to increased susceptibility to cerebral palsy—a pediatric neurodevelopmental disorder thought to be induced by perinatal neuroinflammation—pointing to the importance of COX enzymes in neuroinflammatory pathogenesis and neurodevelopment (69).

Cholesterol, an important component of myelin, is synthesized *de novo* in the brain primarily by astrocytes. Synthesis is high in the developing brain but decreases with age and has a slow turn-over under physiologic conditions (72, 73). Cholesterol is converted to 24-(S)-hydroxycholesterol by CYP46A1, which, in the healthy brain, is expressed in neurons. However, in the presence of neuroinflammation, microglia overexpress CYP46A1, indicating that microglia contribute to cholesterol elimination (74). M1 microglia are known to be associated with the formation of cholesterol-rich lipid droplets (LDs) (75). LDs contain neutral lipids such as cholesterol esters (CE), triacylglycerols (TG), diacylglycerols, phospholipids, and cholesteryl esters. In the brain, LDs are mainly composed of CE and TG. The formation of LDs is regulated by multiple factors including cholesterol synthesis, elimination, and cholesterol efflux from LDs (76). LDs are formed through the uptake of lipids from plasma lipoproteins by specialized endocytic receptors, including scavenger receptors (SR) and low density lipoprotein receptor (LDLR). Both SR CD36 (a fatty acid translocase) and LDLR are upregulated in activated microglia (77–79). Cholesterol efflux from LDs is also important for lipid metabolism in activated microglia. Cholesterol efflux from LDs is mediated by ATP-binding cassette transporter A1 (ABCA1) and ABCG1 (80). The expression of these transporters can be induced by inflammatory cytokines such as IL-6 and TNFα, suggesting that cholesterol efflux from LDs may be enhanced during the activation process. The cholesterol-breakdown product 7-ketocholesterol (7KC) is upregulated in the brains and CSF of patients with multiple sclerosis and propagates a maladaptive pro-inflammatory microglial phenotype through PARP-1 (36, 81). Buildup of 7KC is likely associated with ineffective microglial cholesterol metabolism (36). The balance between cholesterol intake and efflux may play a major role in the pathogenesis and progression of neuroinflammation.

Other lipid-derived metabolites important for secondary injury in TBI may include the endocannabinoids, which activate cannabinoid receptors (82). Cannabinoid receptor 1 is expressed in the brain whereas cannabinoid receptor 2 is expressed mainly on immune cells and tissues. The two main endocannabinoids are 2-arachidonylglycerol (2-AG) and N-arachidonoyl-ethanolamine (AEA), which have locally neuroprotective effects. However, this neuroprotection is short-lived, as both 2-AG and AEA are quickly enzymatically degraded into other modulators of inflammation, including arachidonic acid (82). Katz et al. (82) studied inhibitors of 2-AG and AEA degradation in a rat fluid percussion model of TBI and found that a selective inhibitor of 2-AG degradation improved neurologic and behavioral function and decreased microglial activation. Rats treated with an AEA degradation inhibitor also had decreased microglial activation but did not display significant functional benefits (82). Similarly, the Y. Zhang group has shown anti-inflammatory and neuroprotective benefits of WWL70, an inhibitor of microglial 2-AG hydrolysis, in animal models of TBI, multiple sclerosis, and neuropathic pain (83–85). WWL70 treatment in a mouse model of TBI improved motor coordination and working memory performance, reduced lesion volume, and decreased expression of COX-2 (84). Modulation of the endocannabinoid system and other lipid-derivatives may be useful in treating neuroinflammation.

### Amino Acid Metabolism

Amino acids are an alternative source of energy for certain cells with gluconeogenesis capabilities. Metabolism of amino acids is especially important in the brain because many amino acids act as neurotransmitters. Glutamate is the principal excitatory neurotransmitter of the brain. At high concentrations, glutamate causes neurotoxicity by excessive stimulation of its receptors, including NMDA and kainate receptors. Under normal conditions, astrocytes and microglia strictly regulate glutamate homeostasis, but after TBI, massive glutamate efflux and significant decrease in astrocyte glutamate transporter-1 (GLT-1/EAAT2) cause excitatory cell death (86). Proinflammatory microglia also contribute to releasing large amounts of glutamate as a result of dysregulated oxidative burst and lipid peroxidation (87, 88).

The glutamate-glutamine cycle is well-described (89) but how glutamine metabolism impacts microglial phenotype expression is a much newer concept (Figure 1). Microglia consume glutamine as alternative fuel in the absence of glucose (27). Studies of hypoglycemic mice and brain slices maintained in a completely aglycemic environment have shown that microglial morphology and motility remain unaffected by reduced extracellular glucose for up to 90 minutes (27, 35). Microglia are able to maintain normal function by carrying out oxidative phosphorylation with glutamine. Glutamine enters the microglia through transporters SLC1A5 and SLC38A1 and is converted into glutamate in the mitochondria via glutaminase (Figure 1). The glutamate is metabolized to α-ketoglutarate, which enters the TCA cycle (27). Blocking glutaminolysis in microglia with epigallocatechin gallate or R162 (inhibitors of glutamate-to-α-ketoglutarate metabolism) leads to a decrease in NAD(P)H (35). Blocking glutaminolysis, even without removing glucose, also causes microglia to adopt an amoeboid morphology, reduces motility, and reduces microglial damage response (35).

Microglia play an important role in glutamate metabolism after TBI by ameliorating glutamate-induced neurotoxicity. Microglia clear excess glutamate via glial glutamate transporters such as GLAST/EAAT1, GLT-1/EAAT2 and EAAC1 (90–92), thereby protecting neurons from excitotoxicity. Loss of GLAST and GLT-1 expression on microglia and astrocytes worsens secondary inflammatory damage (90, 91). Another receptor of interest is metabotropic glutamate receptor 5 (mGluR5). A G-protein-coupled receptor expressed in the brain cortex (93), mGluR5, responds to glutamate by modulating microglia to a predominantly anti-inflammatory phenotype (94, 95). mGluR5 agonists have been shown to reduce secondary brain injury after TBI in rats (96). Even delayed activation of mGluR5 1 month after injury helps to attenuate ongoing neuroinflammation and degeneration (97). Increased levels of extracellular glutamate after TBI also have been observed to correlate directly with microglial activation (98). Large amounts of extracellular glutamate activate microglia through NMDA receptors, causing microglia to secrete more glutamate, and eventually impairing mitochondrial respiration. D-cycloserine—an NMDA receptor antagonist, and melatonin—a kainate receptor antagonist, have shown benefit in decreasing microglial activation and enhancing recovery in rodent models of TBI (88, 99).

Tryptophan metabolites also contribute to excitotoxicity. Levels of quinolinic acid, a tryptophan-derived NMDA agonist, rise in the CSF after TBI and correlate directly with mortality in humans (100–102). Tryptophan is metabolized to kynurenine by indoleamine 2,3 dioxygenase 1 (IDO1) in neurons, astrocytes, microglia, and infiltrating macrophages (Figure 2). Kynurenine is, in turn, metabolized into neuroprotective kynurenic acid by kynurenine aminotransferase or into neurotoxic quinolinic acid by kynurenine 3-monooxygenase (103). Modulating the relative activity of neuroprotective kynurenine aminotransferase vs. neurotoxic kynurenine 3-monooxygenase may be beneficial for attenuating inflammation. Tryptophan is also the precursor for neurotransmitters serotonin and melatonin, which are important in regulating mood and sleep (104). Our group has shown that CCI in infant rabbits upregulates IDO1 in microglia, acutely increases kynurenine levels, and decreases serotonin and melatonin at later time points (104). These findings point to a link between microglial activation, tryptophan metabolism, and the long-term sequelae of mood and sleep dysregulation after pediatric TBI.

Arginine metabolism has also been shown to play a key role in the immune function of macrophages and microglia. Arginine is metabolized either through the nitric oxide synthase pathway (seen in M1 macrophages/microglia) to form NO or through the arginase pathway (upregulated in M2 macrophages/microglia). Patients with severe TBI were found to have decreased circulating L-arginine (105) and supplementation with arginine-rich peptides has been shown to have neuroprotective effects in animal models after TBI (106).

## Discussion

Microglial metabolism is a relatively understudied area that can influence both normal development and response to injury. The microglial response, activation, and dysregulation that ensue after pediatric TBI causes inflammatory damage and metabolic changes that can impact brain recovery and maturation thereafter. Understanding the interaction between the metabolic changes that take place in activated microglia, and how these changes affect neighboring astrocytes and neurons, is crucial for limiting inflammation-induced secondary injury (Box 1). If we can understand mechanistically how metabolic changes within microglia affect overall brain energy and metabolism, we can potentially fine-tune metabolic support to diminish the dysregulated cycle of inflammatory damage and improve neurologic outcome.

Box 1 **Future Questions and Areas of Research**

- Do microglia have metabolic plasticity after injury and how do they adapt based on developmental age and pathology?
- What are metabolic pathways that support active functions of microglia (e.g., phagocytosis, mobility, etc.) especially when conventional glucose metabolism in astrocytes and neurons is dysfunctional?
- How does glucose metabolism in microglia relative to other brain cells (astrocytes and neurons) change over time after injury in the immature brain?
- Do infiltrating immune cells have distinct metabolic characteristics that differ from brain microglial cells?
- Do metabolic shifts in brain infiltrating immune cells contribute toward repair or propagate injury after TBI?
- Can we manipulate microglial metabolism and influence microglial phenotype?
- Can microglial metabolism be manipulated as a therapeutic target to enable repair and recovery after pediatric TBI?

**Figure 3:**
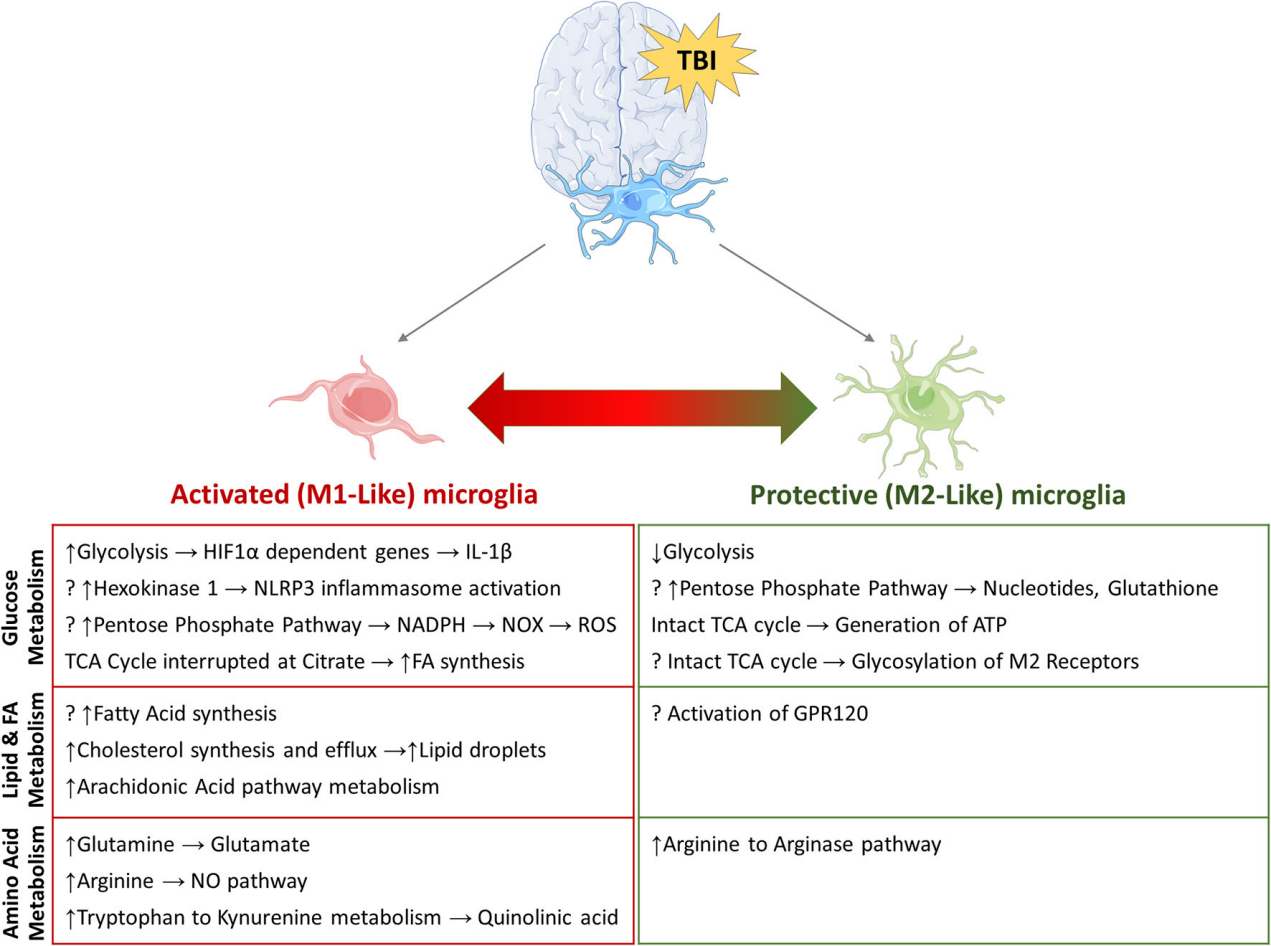
Potential metabolic pathways involved in re-programming of activated microglia. Summary of pathways that have been implicated in microglial re-programming after TBI. (?) indicates potential pathways/mechanisms which have not been elucidated in microglia to date.

This review highlights the vast scope of metabolic pathways that influence microglial function and are influenced by microglial activation after TBI (Figure 3). Manipulating microglial metabolism as a treatment option to modulate the inflammatory response would be a novel mechanism of promoting recovery. Targeted delivery of drugs to manipulate microglial metabolism at a subcellular level using drug-conjugated dendrimer nanoparticles is one such emerging cell-specific therapy. A better understanding of the metabolic pathways that modulate microglial function during normal brain development and following injury is crucial for development of targeted therapies.

## Author Contributions

All authors contributed to the writing of the manuscript under the supervision and guidance of SS and SK. Figures were created by AS.

## Conflict of Interest

The authors declare that the research was conducted in the absence of any commercial or financial relationships that could be construed as a potential conflict of interest.
